# Genomic insights into *Leminorella grimontii* and its chromosomal class A GRI β-lactamase

**DOI:** 10.1007/s10096-024-04888-7

**Published:** 2024-07-03

**Authors:** Claudia Aldeia, Edgar I. Campos-Madueno, Andrea Endimiani

**Affiliations:** 1 Institute for Infectious Diseases (IFIK), University of Bern, Friedbühlstrasse 25, Bern, CH-3001 Switzerland; 2 Graduate School of Cellular and Biomedical Sciences, University of Bern, Bern, Switzerland

**Keywords:** *Leminorella*, ESBL, β-lactamases, GRI-1, Genome, *Zophobas*, Sequencing

## Abstract

*Leminorella grimontii* strain LG-KP-E1-2-T0 was isolated from *Zophobas morio* larvae. It showed a susceptibility phenotype compatible with the expression of an inducible extended-spectrum β-lactamase. The presence of a chromosomal *bla* gene encoding for the class A GRI-1 β-lactamase was revealed by whole-genome sequencing. GRI-1 shared the highest amino acid identity with RIC-1 and OXY-type β-lactamases (76–80%). Analysis of six further publicly-available *L. grimontii* draft genomes deposited in NCBI revealed that *bla*_GRI−1_ was always present. Core-genome analysis indicated that LG-KP-E1-2-T0 was unique from other strains. We provided the first complete genome of *L. grimontii* and new insights on its chromosomal β-lactamases.

**Supplementary Information:**

The online version contains supplementary material available at 10.1007/s10096-024-04888-7.

## Introduction

*Leminorella* spp. are Gram-negative bacteria belonging to the order of Enterobacterales and the family *Budviciaceae* [1]. So far, the *Leminorella* genus includes three taxa: *L. grimontii*, *L. richardii* and *Leminorella* sp. strain 3 [2]. Among them, *L. grimontii* is the most frequently reported in humans [3, 4]. This species has been isolated in stool samples and identified as responsible for spontaneous peritonitis and neonatal sepsis [2–4]. However, no complete genomes of *L. grimontii* are currently available in the NCBI database.

With the exception of carbapenems, most *Leminorella* spp. strains are resistant to β-lactams, but susceptible to β-lactam/β-lactamase inhibitor combinations (βL/βLIC) [5–7]. Therefore, an extended-spectrum β-lactamase (ESBL)-like activity was suggested [6, 7]. In particular, Philippon A et al. (2016) indicated that *L. grimontii* produces GRI-1, a chromosomal class A β-lactamase with a 2be spectrum (UniProt: A4FRA6; GenBank: AM422900.1) [8]. Moreover, two *L. grimontii* protein sequences (WP_027275480.1 and WP_261832807.1) were deposited in 2022 on the NCBI as “class A β-lactamase”. Nevertheless, no *bla*_GRI_ exists in the Reference Gene Catalog of NCBI (https://www.ncbi.nlm.nih.gov/pathogens/refgene/#).

Here, we describe the first complete genome sequence of *L. grimontii*. The strain was isolated from the larvae of the darkling beetle *Zophobas morio* and carried a chromosomally-located *bla*_GRI_ gene.

## Materials and methods

### Isolation and species identification (ID)

*L. grimontii* strain LG-KP-E1-2-T0 was isolated from the homogenized tissues of *Z. morio* larvae plated on ChromID^®^ ESBL agar (bioMérieux). Larvae were acquired from a Swiss pet retailer in 2023 during an ongoing project [9]. Bacterial species ID was carried out using the MALDI-TOF MS (Bruker; FlexControl v3.4 [build 135.14]).

### Phenotypic testing

Antimicrobial susceptibility tests (ASTs) were performed by broth microdilution using the Sensititre™ GNX2F and ESB1F panels (Thermo Fisher Scientific). To detect ESBL(s) production, combination disk testing (CDT) was performed on Mueller-Hinton agar (MHA; Oxoid) with the EUCAST ESBL Disk kit (Liofilchem). The double-disk synergy test (DDST) and induction assays with cefoxitin and imipenem disks were also conducted on MHA (see Figure S1). AST and phenotypic test results were interpreted according to the current EUCAST criteria [10, 11].

### Whole-genome sequencing (WGS)

Genomic DNA was isolated using the Invitrogen™ PureLink™ Microbiome DNA purification kit (Thermo Fisher Scientific) [12, 13]. Purity and gDNA quantification were determined by Nanodrop™ and Qubit™ 3 (Thermo Fisher Scientific). Short-read WGS was performed using the Illumina NovaSeq 6000 sequencer, while long-read WGS was done with the Oxford Nanopore MinION [Oxford Nanopore Technologies (ONT)]. The Rapid Barcoding Kit SQK-RBK004 was used to generate long-read sequencing libraries, which were loaded on a flow cell FLO-MIN 106D R9.4.1 (ONT), and sequenced for 48 h. Short-read data were preprocessed with Trimmomatic v0.36 to remove adapters [14, 15]. The preprocessing of ONT raw data, which includes adapter-trimming and quality filtering, was performed using Porechop v0.2.4 and Filtlong v0.2.1 (parameters: minimum read length of 1-kb and 1,000,000-kb target bases), respectively. Unless indicated otherwise, all bioinformatic analyses were conducted with default parameters. Genome assembly was done with the Unicycler v.0.4.8 hybrid pipeline, followed by coverage estimation using QualiMap v2.2.2 [16, 17].

### Genome characterization

The assembled genome underwent screening using the Center for Genomic Epidemiology (CGE; https://www.genomicepidemiology.org/) and the NCBI AMRFinder (https://github.com/ncbi/amr) databases. Antimicrobial resistance genes (ARGs) were identified using the ResFinder v4.5.0 and the AMRFinder Plus v3.12.8 tools, while plasmid prediction was conducted with PlasmidFinder v2.1 (parameters: 70% threshold identity, 60% minimum length) [18–20]. Putative *bla* genes were screened using the NCBI BLASTn. Insertion sequences (IS) were identified with ISFinder (https://isfinder.biotoul.fr/). Genome-based taxonomy was determined using the Type (Strain) Genome Server (TYGS), while JSpeciesWS was used to determine the average nucleotide identity based on BLASTn (ANIb) [21, 22]. Genome annotation was done automatically with the NCBI Prokaryotic Genome Annotation Pipeline (PGAP) (method: best-placed reference protein set; GeneMarkS-2 + v6.6) [23].

### Core-genome phylogeny

Six *L. grimontii* draft genomes (5 composed of contigs and 1 of scaffolds) available in the NCBI database (retrieved on 05.03.2024) were mapped to the complete assembly of LG-KP-E1-2-T0 using the “–ctgs” flag in Snippy v4.4.5 [24, 25]. *L. grimontii* GCA_958349645.1 draft genome was excluded from the study given its small length.

The snippy-core function was then used to generate a core-genome single nucleotide variant (SNV) alignment. ISs were inferred with ISEScan v1.7.2.3 and masked before SNV calling (i.e., core-genome alignment), while Gubbins v2.3.4 was used to filter SNVs from recombinant regions. SNV distances were calculated with snp-dists v0.8.2. A maximum-likelihood phylogenetic tree, rooted to the most divergent strain LG-KP-E1-2-T0, was built with IQ-TREE v2.3.0 (parameters: GTR + ASC, -bb 1000, -alrt 1000), visualized with iTOL v6.9 and annotated with Inkscape v1.3.

### Analysis of GRI amino acid sequences

All *bla* coding sequences (CDS) confirmed by BLASTn as *bla*_GRI_ were extracted from the six *L. grimontii* draft genomes. Subsequently, they were translated into amino acid sequences using Geneious Prime (Biomatters) v2023.2.1 (parameters: genetic code, bacterial, transl_table 11). Furthermore, the three previously deposited proteins A4FRA6, WP_027275480.1 and WP_261832807.1 and the one found in LG-KP-E1-2-T0 were used to generate a multiple sequence alignment with MUSCLE v5.1 in Geneious Prime.

A BLASTp search was performed using the GRI sequence of LG-KP-E1-2-T0 [26]. Best-hit protein sequences were retrieved from the NCBI Bacterial Antimicrobial Resistance Reference Gene Database (https://www.ncbi.nlm.nih.gov/bioproject/313047), along with other representative class A β-lactamase sequences, and subjected to an alignment and phylogenetic inference using the online MAFFT v7 (parameters: size, 40 sequences x 266 sites; model, Jones-Taylor-Thornton (JTT) and Bootstrap resampling, 100; https://mafft.cbrc.jp/alignment/server/index.html). The resulting tree was rooted to TEM β-lactamases, the most phylogenetically distant family (*Table **S1*) and annotated with iTOL v6.9 and Inkscape v1.3, respectively.

## Results and discussion

### Phenotypic testing

The MALDI-TOF MS identified LG-KP-E1-2-T0 as *L. grimontii* (score of 2.36). The strain was resistant to cefotaxime and aztreonam, but susceptible to cefoxitin, ceftriaxone, cefepime, carbapenems and βL/βLIC (Table 1). This phenotype was consistent with the production of an ESBL, as further confirmed by the results of the CDT and DDST assays (*Figure **S1**-A/B*) [10, 11]. Moreover, an inducible phenotype was suspected with the DDST and well-confirmed with the cefoxitin and imipenem assays (*Figure **S1**-C/D*).

**Table 1:** Antimicrobial susceptibility profile of the *L. grimontii* strain LG-KP-E1-2-T0

Antibiotics	MIC (µg/mL), interpretation ^a^
Piperacillin-tazobactam	≤ 8/4, S
Ticarcillin-clavulanate	≤ 16/2, S
Ampicillin	> 16, R
Ceftazidime	≤ 1, S
Ceftazidime-clavulanate	≤ 0.12/4, NA
Cefazolin	> 16, R
Cefoxitin	≤ 4, S
Cephalothin	> 16, NA
Cefpodoxime	> 32, R
Cefotaxime	4, R
Cefotaxime-clavulanate	≤ 0.12/4, NA
Ceftriaxone	≤ 1, S
Cefepime	≤ 1, S
Aztreonam	8, R
Imipenem	≤ 1, S
Meropenem	≤ 1, S
Doripenem	≤ 0.12, S
Ertapenem	≤ 0.25, S
Gentamicin	≤ 1, S
Tobramycin	≤ 1, S
Amikacin	8, S
Ciprofloxacin	≤ 0.25, S
Levofloxacin	≤ 1, S
Colistin	≤ 0.25, S
Polymyxin B	≤ 0.25, NA
Doxycycline	≤ 2, NA
Minocycline	≤ 2, NA
Tigecycline	≤ 0.25, S
Trimethoprim-sulfamethoxazole	≤ 0.5/9.5, S

*Note* R, resistant; S, susceptible; NA, interpretative criteria not available; MIC, minimum inhibitory concentration

^**a**^ Antimicrobial susceptibility was determined using the Sensititre™ GNX2F and ESB1F panels. MICs were interpreted according to the 2024 EUCAST criteria for Enterobacterales [10]

### Genomic features of LG-KP-E1-2-T0

**Fig. 1:**
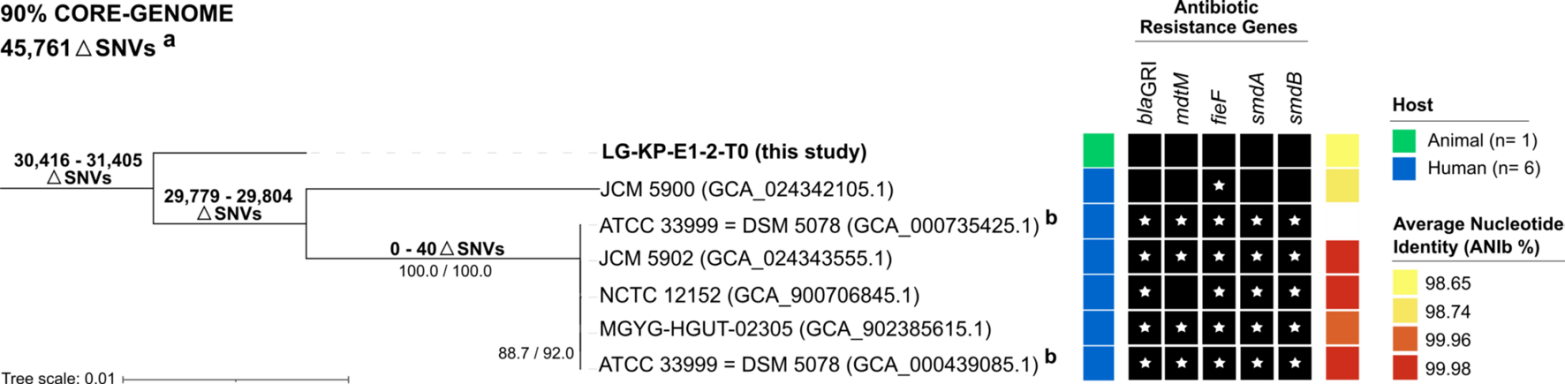
Core genome-based maximum-likelihood phylogenetic tree. ^**a**^ Alignment of all *L. grimontii* genomes (*n* = 7), which resulted in 45,761 SNVs considering 90% of all aligned genomes. ^**b**^*L. grimontii* ATCC 33999 = DSM 5078 (accession numbers GCA_000735425.1 and GCA_000439085.1) represents the deposited NCBI reference genome (release date: 2014) and the TYGS type strain genome used for species identification (release date: 2013), respectively. The tree was rooted to LG-KP-E1-2-T0 as outgroup. Bootstrap support is shown in internal nodes (SH-aLRT ≥ 80% and UFBoot ≥ 95%, respectively). Corresponding GenBank accession numbers are given in parentheses. The tree scale represents the average number of nucleotide substitutions per site. Single nucleotide variants (SNVs) shared among genomes are represented by a ΔSNVs. Color-coded boxes in the columns show (from the left) for each strain: host, presence of antimicrobial resistance genes (ARGs; black square), and its average nucleotide identity (ANIb %) to the NCBI reference genome ATCC 33999 = DSM 5078 (GCA_000735425.1). A star at the center of the column indicates ARGs that are 100% identical to that of the NCBI reference strain

Illumina and Nanopore sequencing runs yielded a total of 10,323,042-bp and 879,684-bp (*N*_50_ = 7,736-bp) reads, respectively, which were used to generate the complete genome of strain LG-KP-E1-2-T0. As a result, a circular 4,335,522-bp chromosome (GC content, 53.8%) with 358.97× coverage was obtained, whereas plasmids (replicons) were not identified [18]. TYGS and JSpeciesWS analysis confirmed that LG-KP-E1-2-T0 belonged to *L. grimontii* [21, 22]. As shown in Fig. 1, LG-KP-E1-2-T0 had an ANIb value of 98.65% to the NCBI reference genome ATCC 33999 = DSM 5078 (GCA_000735425.1).

Core-genome phylogeny resulted in a total of 45,761 SNVs and identified 3 distinct groups (Fig. 1): two groups contained a single strain, either LG-KP-E1-2-T0 or JCM 5900, while the remaining genomes clustered together (△SNVs = 0–40) in a separate group. Moreover, the closest match to LG-KP-E1-2-T0 was the reference genome ATCC 33999 = DSM 5078 (△SNVs = 30,416).

### Resistance genes in LG-KP-E1-2-T0

Within the chromosome of LG-KP-E1-2-T0, an 888-bp *bla* gene was identified in the PGAP annotation between positions 3,668,018-bp and 3,668,905-bp. The *ampR* gene (LysR family transcriptional regulator) was also found upstream [24]; such element has been previously associated to inducible class A ESBLs (e.g., SFO-1) [27].

ARG detection using nucleotide and protein databases of ResFinder and AMRFinder Plus identified a *bla* CDS most similar to *bla*_OXY-3-1_ (75.86% identity, 89.39% coverage) and OXY-10-1 (78.98% identity, 100% coverage), respectively (*data not shown*). Using the NCBI BLASTn search, the putative *bla* CDS of LG-KP-E1-2-T0 showed the highest similarity to the deposited *bla*_GRI_ gene of *L. grimontii* (GenBank: AM422900.1), with 98.99% identity and 100% coverage.

Other resistance mechanisms such as the multidrug efflux pumps MdtM, SmdA, SmdB and the iron efflux transporter FieF (70.50%, 72.71%, 70.36% and 72.58% amino acid identity to AMRFinder protein sequences, respectively) were also detected [20, 28]. Furthermore, no IS elements were found in the neighboring regions of the *bla*_GRI_ that could suggest potential mobilization events [29].

### The GRI-I β-lactamase

**Fig. 2:**
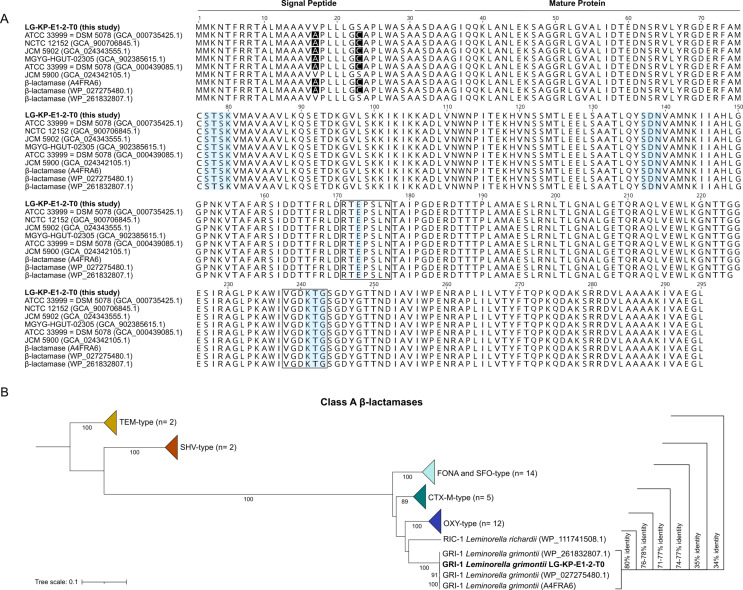
(**A**) Structure-based protein alignment of GRI-1 β-lactamase from *L. grimontii*. The signal peptide and mature protein regions are delineated in black, above the sequence. At substitution site, identical amino acid residues to each other are illustrated in black. Strictly conserved motifs in class A enzymes [SXXK (active site: position 70–73), SDN (position 130–132), E and KTG (positions 166 and 234–236)], subclass A1 [RXEXXLN (position 164–170), VGDKTG (position 231–236)] are shown in light blue and framed in black, respectively. Corresponding GenBank accession numbers are given in parentheses. (**B**) The phylogenetic tree represents the similarity at the amino acid sequence level of 40 representative class A β-lactamases. Bootstrap support (≥ 80%) is displayed below the nodes. The following sequences obtained from the NCBI Bacterial Antimicrobial Resistance Reference Gene Database were considered: TEM-1/-12, SHV-1/-12, CTX-M-1/-8/-15/-40, KLUG-1, OXY-1 (-4, -8, -13, -16 subvariants), OXY-3-1, OXY-5 (-1, -5, -6, -7, -11 subvariants), OXY-7-1, OXY-10-1, FONA-type (variants 1 to 13) and SFO-1. The protein sequences of GRI-1 and RIC-1 corresponding to the accession numbers WP_261832807.1, WP_027275480.1 and WP_111741508.1, were retrieved from the NCBI Reference Sequence, while for GRI-1 (A4FRA6) was retrieved from UniProt. On the right is the amino acid similarity between β-lactamase families and GRI-1 of *L. grimontii*

The *bla*_GRI_ found in LG-KP-E1-2-T0 encoded a protein of 295 amino acid residues with all classic motifs of class A/subclass A1 β-lactamases (Fig. 2A) [8]. This enzyme shared 100% similarity with those from JCM 5900 and WP_261832807.1. In contrast, compared to that of the remaining sequences, the GRI protein of LG-KP-E1-2-T0 differed by two amino acid substitutions (V17A and S23C) in the signal peptide. Since no amino acid substitutions were identified in the leader sequence, we classified all encoded GRI β-lactamases of the *L. grimontii* genomes as GRI-1 [30].

As shown in Fig. 2B and File S1, the peptide alignment performed on 40 representative amino acid sequences revealed a clear separation between GRI-1 and the other families of class A β-lactamases. The encoded RIC-1 chromosomal β-lactamase of *L. richardii* clustered very closely to GRI-1 (~ 80% identity) [8], while OXY, CTX-M, FONA and SFO families displayed 76–78%, 71–77%, 74–77% and 74–75% identity, respectively. In contrast, TEM and SHV families were the most divergent (34% and 35% identity, respectively).

## Conclusions

We provided the first complete genome of *L. grimontii*, an emerging pathogen in the clinical context [3, 5]. The strain was unexpectedly isolated from *Z. morio* larvae [9], though *L. grimontii* was also found in the gut microflora of mosquito and red palm weevil [31, 32].

Our genome comparative analysis, along with the phenotypic confirmatory testing, suggested that all *L. grimontii* express an inducible chromosomally-encoded class A ESBL (GRI-1) with cefotaximase activity. Future studies should be directed at finding possible GRI variants and characterizing the kinetic properties of these enzymes. Moreover, the available complete genome of *L. grimontii* may be useful for larger epidemiological analyses.

### Electronic supplementary material

Below is the link to the electronic supplementary material.

Supplementary Material 1

## Data Availability

LG-KP-E1-2-T0 complete genome assembly was deposited in GenBank under CP146357.1 and with BioProject accession number PRJNA1081762.

## References

[1] Adeolu M, Alnajar S, Naushad S (2016) R SG Genome-based phylogeny and taxonomy of the ‘Enterobacteriales’: proposal for Enterobacterales ord. nov. divided into the families *Enterobacteriaceae*, *Erwiniaceae* fam. nov., *Pectobacteriaceae* fam. nov., *Yersiniaceae* fam. nov., *Hafniaceae* fam. nov., *Morganellaceae* fam. nov., and *Budviciaceae* fam. nov. Int J Syst Evol Microbiol 66 (12):5575–5599 10.1099/ijsem.0.001485 27620848

[2] Hickman-Brenner FW, Vohra MP, Huntley-Carter GP, Fanning GR, Lowery VA 3rd, Brenner DJ, Farmer JJ 3rd (1985) *Leminorella*, a new genus of *Enterobacteriaceae*: identification of *Leminorella grimontii* sp. nov. and *Leminorella richardii* sp. nov. found in clinical specimens. J Clin Microbiol 21(2):234–239 3972991 10.1128/jcm.21.2.234-239.1985 PMC271620

[3] Dalamaga M, Karmaniolas K, Pantelaki M, Daskalopoulou K, Kavatha D, Migdalis I (2006) Spontaneous peritonitis caused by *Leminorella grimontii*. Diagn Microbiol Infect Dis 56(1):83–85 16650952 10.1016/j.diagmicrobio.2006.03.006

[4] Sharma D, Patel A, Soni P, Shastri S, Singh R (2017) *Leminorella* sepsis in very low birth weight neonate as cause of neonatal mortality. J Maternal-Fetal Neonatal Med 30(9):1057–1059 10.1080/14767058.2016.1199678 27279269

[5] Blekher L, Siegman-Igra Y, Schwartz D, Berger SA, Carmeli Y (2000) Clinical significance and antibiotic resistance patterns of *Leminorella* spp., an emerging nosocomial pathogen. J Clin Microbiol 38(8):3036–3038 10921973 10.1128/JCM.38.8.3036-3038.2000 PMC87180

[6] Stone ND, O’Hara CM, Williams PP, McGowan JE Jr., Tenover FC (2007) Comparison of disk diffusion, VITEK 2, and broth microdilution antimicrobial susceptibility test results for unusual species of *Enterobacteriaceae*. J Clin Microbiol 45(2):340–346 17135429 10.1128/JCM.01782-06 PMC1829079

[7] Abbott SL, Lidgard JA, Cheung WK, Obeso MN, Berrada ZL, Janda JM (2012) Expression of ESBL-like activity in infrequently encountered members of the family *Enterobacteriaceae*. Curr Microbiol 64(3):222–225 22139464 10.1007/s00284-011-0057-4

[8] Philippon A, Slama P, Deny P, Labia R (2016) A structure-based classification of class A β-Lactamases, a broadly diverse family of enzymes. Clin Microbiol Rev 29(1):29–57 26511485 10.1128/CMR.00019-15 PMC4771212

[9] Eddoubaji Y, Aldeia C, Campos-Madueno EI, Moser AI, Kundlacz C, Perreten V, Hilty M, Endimiani A (2024) A new *in vivo* model of intestinal colonization using *Zophobas morio* larvae: testing hyperepidemic ESBL- and carbapenemase-producing *Escherichia coli* clones. Frontiers in Microbiology 15 10.3389/fmicb.2024.1381051 PMC11039899 38659985

[10] EUCAST The European Committee on Antimicrobial Susceptibility Testing. Breakpoint tables for interpretation of MICs and zone diameters, version 14.0 (2024) https://www.eucast.org/clinical_breakpoints

[11] EUCAST guidelines for detection (2017) Of resistance mechanisms and specific resistances of clinical and/or epidemiological importance. Version 2.0

[12] Aldeia C, Campos-Madueno EI, Sendi P, Endimiani A (2023) Complete genome sequence of the first colistin-resistant *Raoultella electrica* strain. Microbiol Resource Announcements 12(5):e00047–e00023 10.1128/mra.00047-23 PMC10190570 37014211

[13] Campos-Madueno EI, Aldeia C, Perreten V, Sendi P, Moser AI, Endimiani A (2023) Detection of *bla*_CTX-M_ and *bla*_DHA_ genes in stool samples of healthy people: comparison of culture- and shotgun metagenomic-based approaches. Frontiers in Microbiology 14 10.3389/fmicb.2023.1236208 PMC10501143 37720151

[14] Bolger AM, Lohse M, Usadel B (2014) Trimmomatic: a flexible trimmer for Illumina sequence data. Bioinformatics 30(15):2114–2120 24695404 10.1093/bioinformatics/btu170 PMC4103590

[15] Wick RR, Judd LM, Gorrie CL, Holt KE (2017) Completing bacterial genome assemblies with multiplex MinION sequencing. Microb Genomics 3 (10) 10.1099/mgen.0.000132 PMC5695209 29177090

[16] García-Alcalde F, Okonechnikov K, Carbonell J, Cruz LM, Götz S, Tarazona S, Dopazo J, Meyer TF, Conesa A (2012) Qualimap: evaluating next-generation sequencing alignment data. Bioinformatics 28(20):2678–2679 22914218 10.1093/bioinformatics/bts503

[17] Wick RR, Judd LM, Gorrie CL, Holt KE (2017) Unicycler: resolving bacterial genome assemblies from short and long sequencing reads. PLoS Comput Biol 13(6):e1005595 28594827 10.1371/journal.pcbi.1005595 PMC5481147

[18] Carattoli A, Zankari E, Garcia-Fernandez A, Voldby Larsen M, Lund O, Villa L, Moller Aarestrup F, Hasman H (2014) *In silico* detection and typing of plasmids using PlasmidFinder and plasmid multilocus sequence typing. Antimicrob Agents Chemother 58(7):3895–3903 24777092 10.1128/AAC.02412-14 PMC4068535

[19] Feldgarden M, Brover V, Haft DH, Prasad AB, Slotta DJ, Tolstoy I, Tyson GH, Zhao S, Hsu CH, McDermott PF, Tadesse DA, Morales C, Simmons M, Tillman G, Wasilenko J, Folster JP, Klimke W (2019) Validating the AMRFinder Tool and Resistance Gene Database by using Antimicrobial Resistance genotype-phenotype correlations in a Collection of isolates. Antimicrob Agents Chemother 63 (11) 10.1128/AAC.00483-19 PMC6811410 31427293

[20] Bortolaia V, Kaas RS, Ruppe E, Roberts MC, Schwarz S, Cattoir V, Philippon A, Allesoe RL, Rebelo AR, Florensa AF, Fagelhauer L, Chakraborty T, Neumann B, Werner G, Bender JK, Stingl K, Nguyen M, Coppens J, Xavier BB, Malhotra-Kumar S, Westh H, Pinholt M, Anjum MF, Duggett NA, Kempf I, Nykasenoja S, Olkkola S, Wieczorek K, Amaro A, Clemente L, Mossong J, Losch S, Ragimbeau C, Lund O, Aarestrup FM (2020) ResFinder 4.0 for predictions of phenotypes from genotypes. J Antimicrob Chemother 75(12):3491–3500 32780112 10.1093/jac/dkaa345 PMC7662176

[21] Meier-Kolthoff JP, Göker M (2019) TYGS is an automated high-throughput platform for state-of-the-art genome-based taxonomy. Nat Commun 10(1):2182 31097708 10.1038/s41467-019-10210-3 PMC6522516

[22] Richter M, Rosselló-Móra R, Oliver Glöckner F, Peplies J (2015) JSpeciesWS: a web server for prokaryotic species circumscription based on pairwise genome comparison. Bioinformatics 32(6):929–931 26576653 10.1093/bioinformatics/btv681 PMC5939971

[23] Tatusova T, DiCuccio M, Badretdin A, Chetvernin V, Nawrocki E, Zaslavsky L, Lomsadze A, Pruitt K, Borodovsky M, Ostell J (2016) NCBI prokaryotic genome annotation pipeline. Nucleic Acids Res 44:gkw569 10.1093/nar/gkw569 PMC5001611 27342282

[24] Kundlacz C, Aldeia C, Eddoubaji Y, Campos-Madueno EI, Endimiani A (2024) A new OCH β-lactamase from a *Brucella pseudintermedia* (*Ochrobactrum pseudintermedium*) strain isolated from *Zophobas morio* larvae. J Glob Antimicrob Resist 36:65–69 10.1016/j.jgar.2023.12.012 38128729

[25] Campos-Madueno EI, Aldeia C, Sendi P, Endimiani A (2023) *Escherichia ruysiae* May serve as a Reservoir of Antibiotic Resistance genes across multiple settings and regions. Microbiol Spectr 11(4):e0175323 10.1128/spectrum.01753-23 PMC10434276 37318364

[26] Altschul SF, Gish W, Miller W, Myers EW, Lipman DJ (1990) Basic local alignment search tool. J Mol Biol 215(3):403–410 2231712 10.1016/S0022-2836(05)80360-2

[27] Matsumoto Y, Inoue M (1999) Characterization of SFO-1, a plasmid-mediated inducible class a β-lactamase from *Enterobacter cloacae*. Antimicrob Agents Chemother 43(2):307–313 10.1128/aac.43.2.307 PMC89069 9925524

[28] Feldgarden M, Brover V, Gonzalez-Escalona N, Frye JG, Haendiges J, Haft DH, Hoffmann M, Pettengill JB, Prasad AB, Tillman GE, Tyson GH, Klimke W (2021) AMRFinderPlus and the reference gene catalog facilitate examination of the genomic links among antimicrobial resistance, stress response, and virulence. Sci Rep 11(1):12728 34135355 10.1038/s41598-021-91456-0 PMC8208984

[29] Siguier P, Perochon J, Lestrade L, Mahillon J, Chandler M (2006) ISfinder: the reference centre for bacterial insertion sequences. Nucleic Acids Res 34(suppl1):D32–D36 16381877 10.1093/nar/gkj014 PMC1347377

[30] Bradford PA, Bonomo RA, Bush K, Carattoli A, Feldgarden M, Haft DH, Ishii Y, Jacoby GA, Klimke W, Palzkill T, Poirel L, Rossolini GM, Tamma PD, Arias CA (2022) Consensus on β-Lactamase nomenclature. Antimicrob Agents Chemother 66(4):e00333–e00322 35380458 10.1128/aac.00333-22 PMC9017354

[31] Farah Nadiah R, Norefrina Shafinaz M, Nurul Wahida O (2018) Preliminary study of gut bacterial abundance in *Rhynchophorus ferrugineus* (Coleoptera: *Dryophthoridae*) fed on different diets. Serangga 23:126–138

[32] Rani A, Sharma A, Rajagopal R, Adak T, Bhatnagar RK (2009) Bacterial diversity analysis of larvae and adult midgut microflora using culture-dependent and culture-independent methods in lab-reared and field-collected *Anopheles stephensi*-an Asian malarial vector. BMC Microbiol 9(1):96 19450290 10.1186/1471-2180-9-96 PMC2698833

