# Supplementary material for: Genomic insights into Leminorella grimontii and its chromosomal class A GRI β-lactamase
Source: Eur J Clin Microbiol Infect Dis. 2024 Jul 3;43(9):1855–60. doi: 10.1007/s10096-024-04888-7 (PMC11349772; doi:10.1007/s10096-024-04888-7)
Supplement: Supplementary file 1 — Supplementary Material 1 [file 10096_2024_4888_MOESM1_ESM.pptx]

## Slide 1
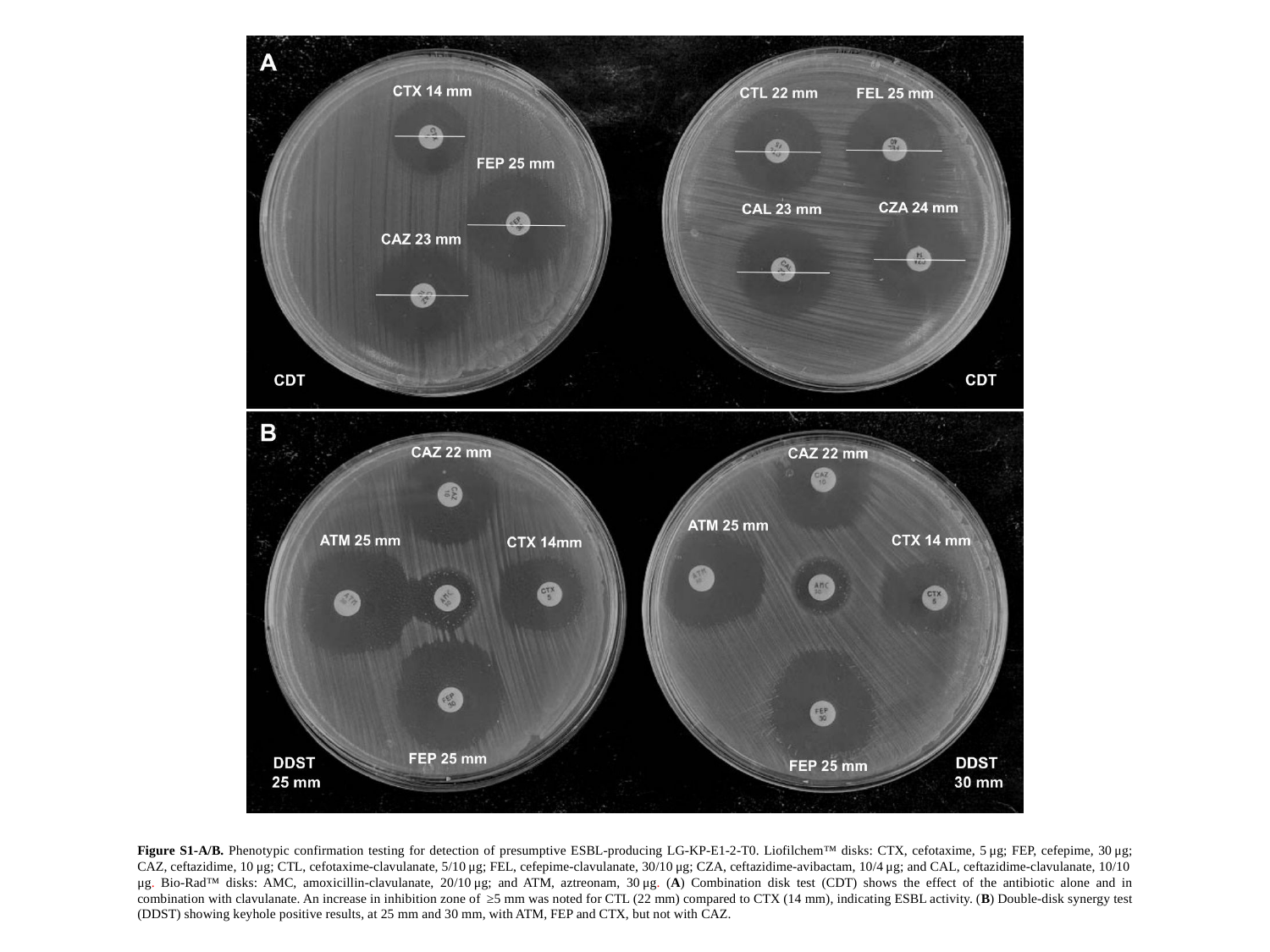

Figure S1-A/B. Phenotypic confirmation testing for detection of presumptive ESBL-producing LG-KP-E1-2-T0. Liofilchem™ disks: CTX, cefotaxime, 5 μg; FEP, cefepime, 30 μg; CAZ, ceftazidime, 10 μg; CTL, cefotaxime-clavulanate, 5/10 μg; FEL, cefepime-clavulanate, 30/10 μg; CZA, ceftazidime-avibactam, 10/4 μg; and CAL, ceftazidime-clavulanate, 10/10 μg. Bio-Rad™ disks: AMC, amoxicillin-clavulanate, 20/10 μg; and ATM, aztreonam, 30 μg. (A) Combination disk test (CDT) shows the effect of the antibiotic alone and in combination with clavulanate. An increase in inhibition zone of  ≥5 mm was noted for CTL (22 mm) compared to CTX (14 mm), indicating ESBL activity. (B) Double-disk synergy test (DDST) showing keyhole positive results, at 25 mm and 30 mm, with ATM, FEP and CTX, but not with CAZ.

## Slide 2
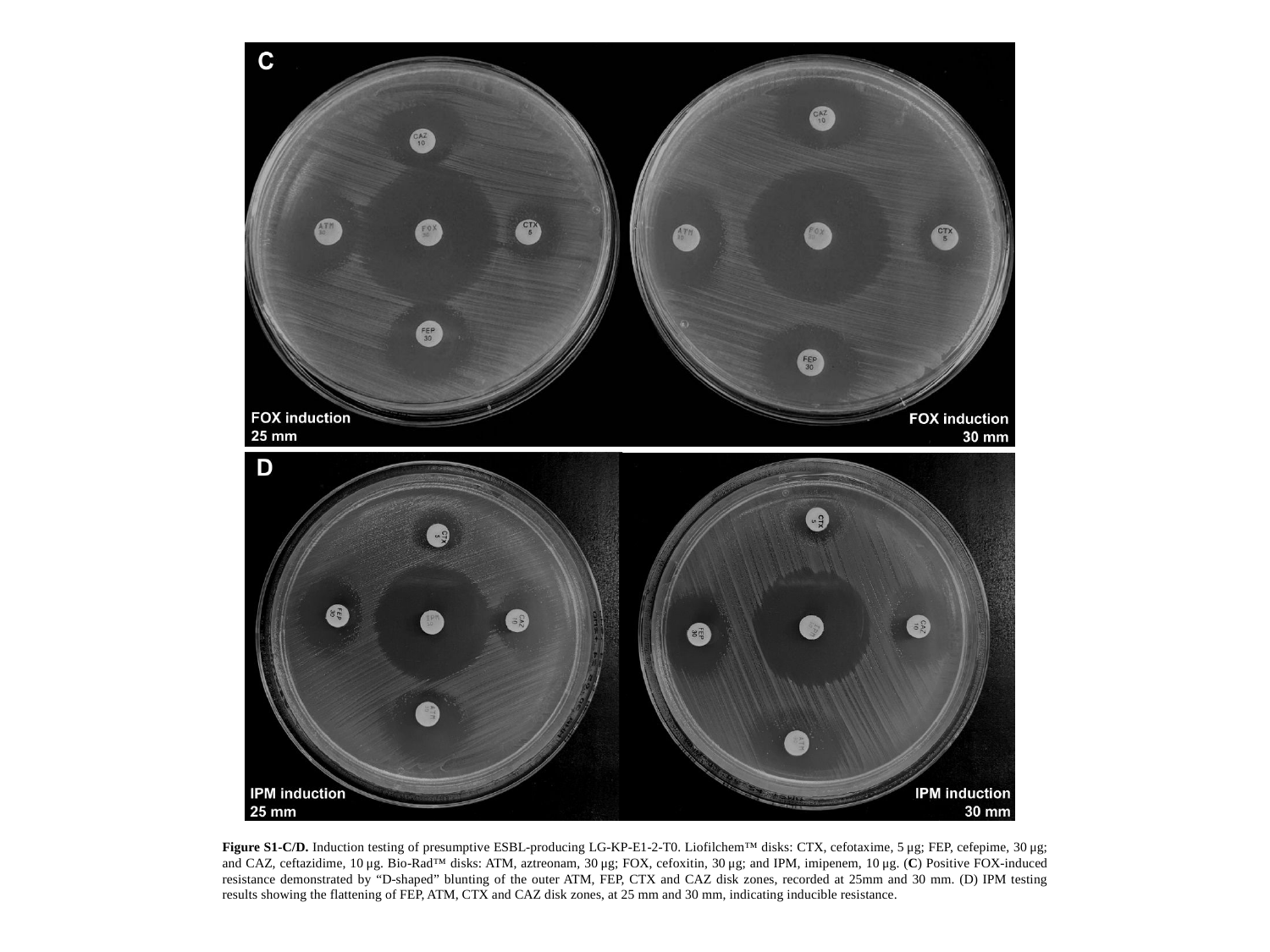

Figure S1-C/D. Induction testing of presumptive ESBL-producing LG-KP-E1-2-T0. Liofilchem™ disks: CTX, cefotaxime, 5 μg; FEP, cefepime, 30 μg; and CAZ, ceftazidime, 10 μg. Bio-Rad™ disks: ATM, aztreonam, 30 μg; FOX, cefoxitin, 30 μg; and IPM, imipenem, 10 μg. (C) Positive FOX-induced resistance demonstrated by “D-shaped” blunting of the outer ATM, FEP, CTX and CAZ disk zones, recorded at 25mm and 30 mm. (D) IPM testing results showing the flattening of FEP, ATM, CTX and CAZ disk zones, at 25 mm and 30 mm, indicating inducible resistance.
